# The Effects of Neoadjuvant Chemoradiation in Locally Advanced Rectal Cancer—The Impact in Intratumoral Heterogeneity

**DOI:** 10.3389/fonc.2019.00974

**Published:** 2019-09-27

**Authors:** Fabiana Bettoni, Cibele Masotti, Bruna R. Corrêa, Elisa Donnard, Filipe F. dos Santos, Guilherme P. São Julião, Bruna B. Vailati, Angelita Habr-Gama, Pedro A. F. Galante, Rodrigo O. Perez, Anamaria A. Camargo

**Affiliations:** ^1^Hospital Sírio Libanês, São Paulo, Brazil; ^2^Instituto Angelita and Joaquim Gama, São Paulo, Brazil; ^3^Ludwig Institute for Cancer Research, São Paulo, Brazil

**Keywords:** neoadjuvant therapy, rectal cancer, intratumoral heterogeneity, clonal evolution, therapy resistance

## Abstract

**Purpose:** Intratumoral genetic heterogeneity (ITGH) is a common feature of solid tumors. However, little is known about the effect of neoadjuvant chemoradiation (nCRT) in ITGH of rectal tumors that exhibit poor response to nCRT. Here, we examined the impact of nCRT in the mutational profile and ITGH of rectal tumors and its adjacent irradiated normal mucosa in the setting of incomplete response to nCRT.

**Methods and Materials:** To evaluate ITGH in rectal tumors, we analyzed whole-exome sequencing (WES) data from 79 tumors obtained from The Cancer Genome Atlas (TCGA). We also compared matched peripheral blood cells, irradiated normal rectal mucosa and pre and post-treatment tumor samples (PRE-T and POS-T) from one individual to examine the iatrogenic effects of nCRT. Finally, we performed WES of 7 PRE-T/POST-T matched samples to examine how nCRT affects ITGH. ITGH was assessed by quantifying subclonal mutations within individual tumors using the Mutant-Allele Tumor Heterogeneity score (MATH score).

**Results:** Rectal tumors exhibit remarkable ITGH that is ultimately associated with disease stage (MATH score stage I/II 35.54 vs. stage III/IV 44.39, *p* = 0.047) and lymph node metastasis (MATH score N0 35.87 vs. *N*+ 45.79, *p* = 0.026). We also showed that nCRT does not seem to introduce detectable somatic mutations in the irradiated mucosa. Comparison of PRE-T and POST-T matched samples revealed a significant increase in ITGH in 5 out 7 patients and MATH scores were significantly higher after nCRT (median 41.7 vs. 28.8, *p* = 0.04). Finally, we were able to identify a subset of “enriched mutations” with significant changes in MAFs between PRE-T and POST-T samples. These “enriched mutations” were significantly more frequent in POST-T compared to PRE-T samples (92.9% vs. 7.1% *p* < 0.00001) and include mutations in genes associated with genetic instability and drug resistance in colorectal cancer, indicating the expansion of tumor cell subpopulations more prone to resist to nCRT.

**Conclusions:** nCRT increases ITGH and may result in the expansion of resistant tumor cell populations in residual tumors. The risk of introducing relevant somatic mutations in the adjacent mucosa is minimal but non-responsive tumors may have potentially worse biological behavior when compared to their untreated counterparts. This was an exploratory study, and due to the limited number of samples analyzed, our results need to be validated in larger cohorts.

## Introduction

Neoadjuvant chemoradiotherapy (nCRT) is one of the preferred treatment strategies for locally advanced rectal cancer (1, 2). In addition to providing improved local disease control (particularly for patients with high-risk features for local recurrence), nCRT may allow the opportunity for organ-preservation among patients with complete clinical response (cCR). However, treatment of patients with low-risk for local recurrence with nCRT, for the sole purpose of organ-preservation, may result in significant detrimental functional and biological consequences among patients who do not achieve a cCR and still need radical surgery.

Intratumoral genetic heterogeneity (ITGH) was first described in the early 1980's (3). However, only recently, the full extent and the functional implications of ITGH have been appreciated (4). ITGH increases phenotypic variation and is currently seen as a critical mechanism underlying disease progression and therapeutic failure (5, 6). We and others have recently characterized the clonal architecture of locally advanced rectal tumors through multi-region whole-exome sequencing (WES). We demonstrated that non-treated rectal tumors exhibit a complex clonal architecture and significant, ITGH with 27–97% of exonic somatic mutations shared among all regions of an individual's tumor and with a mutant allele frequency (MAF) correlation between disparate tumor regions ranging from *R*^2^ = 0.69–0.96 (7, 8). However, in these studies ITGH, was determined using a small number of tumors, and the effect of nCRT in shaping the mutational landscape and clonal architecture of rectal cancer was not addressed. Ultimately, tumors that do not respond completely to nCRT may acquire novel mutations and/or harbor selected tumor cells subpopulations compared to their baseline counterparts, leading to increased ITGH. Here, we evaluated ITGH in untreated rectal tumors and examined its association with disease stage and presence of lymph node metastasis. Also, we analyzed the impact of nCRT in the mutational landscape and ITGH of rectal tumors with incomplete response to nCRT and searched for somatic mutations introduced by nCRT in the adjacent normal irradiated mucosa.

## Materials and Methods

### TCGA Data

To evaluate ITGH in rectal tumors and determine its association with disease stage and presence of lymph node metastasis, we analyzed whole-exome sequencing (WES) data from 79 rectal tumors from The Cancer Genome Atlas (TCGA) colorectal cohort (9, 10). Clinical, pathological and mutational data for all 79 rectal tumors are provided in Supplementary Table 1.

### Rectal Cancer Patients and nCRT

Consecutive patients with rectal cancer (adenocarcinoma biopsy-proven), located no more than 7 cm from the anal verge, and treated at the Angelita & Joaquim Gama Institute between 2007 and 2010, were eligible for the study. Only patients undergoing neoadjuvant chemoradiation were recruited for the study. Inclusion criteria included tumors with cT3/T4 or cN+ disease by radiological staging using magnetic resonance (MR) or endorectal ultrasound. Additionally, patients with cT2N0 otherwise considered for abdominal perineal excision or ultra-low anterior resections were also referred for neoadjuvant chemoradiation and included in the study. Patients with metastatic disease were excluded from the study. Patients with clinical and radiological findings consistent with cCR were also excluded from the present study. Only patients with ≥10% residual cancer cells in the final pathological assessment were included in an attempt to avoid contamination of “incomplete responders” with “near-complete responders” that could eventually develop complete response if longer resting intervals had been used (Table 1). Macrodissection of tumor regions was performed whenever necessary prior to DNA extraction to increase sample purity. Tumor sections were required to contain at least 80% tumor cell nuclei with <20% necrosis for inclusion in the study. We have randomly selected one of the patients for the analysis of the nCRT effect on the normal rectal mucosa. Baseline staging and assessment of patients included digital rectal examination (DRE), proctoscopy and high-resolution MR. All patients underwent long-course chemoradiation therapy as described previously (11).

**Table 1:** Clinical and pathological data of rectal cancer patients submitted to nCRT.

**Patient**	**Tumor size (cm)**	**Distance from anal verge (cm)**	**Baseline stage**	**Type of operation**	**Pathology**	**TRG (% tumor)**
PT01	5	5	cT3N0M0	LAR	ypT3N0	80
PT02	4	6	cT3N1M0	LAR	ypT2N1	80
PT03	4	7	cT3N1M0	LAR	ypT2N0	90
PT04	3	5	cT2N1M0	LAR	ypT2N2	30
PT05	5	4	cT2N1M0	APR	ypT3N0	70
PT06	5	6	cT2N1M0	LAR	ypT2N0	90
PT07	4	4	cT3N0M0	LAR	ypT3N1	80

*LAR, low anterior resection; APR, abdominal perineal resection; TRG, tumor regression grade*.

### Assessment of Tumor Response

All patients were assessed for tumor response after at least 12 weeks from the last day of nCRT completion. Assessment of tumor response was performed with DRE, proctoscopy and MR. Patients with incomplete clinical response (clinical or radiological) were referred to immediate radical surgery.

### Tumor and Blood Samples

Tumor samples were collected at diagnosis (PRE-T samples) and during surgical removal of the residual tumor (POST-T samples). Tumor-adjacent normal colonic mucosa exposed to nCRT (Nrx) was also collected from one patient immediately after tumor resection. Peripheral blood cells (BC) were collected from all patients before nCRT. This study was approved by the Ethics Committee of Hospital Alemão Oswaldo Cruz, São Paulo, Brazil (reference number 19/08) and was conducted in accordance with the Declaration of Helsinki. Patients provided written informed consent for tumor sample collection and study participation. Samples were processed as described in Supplementary Material.

### Whole Exome Sequencing (WES)

Whole-exome libraries were prepared using SureSelect Human All Exon Target Enrichment kit (Agilent Technologies, Santa Clara, CA) and sequences were generated on a 5500xl SOLiD sequencing platform (Thermo-Fisher Scientific, Waltham, MA). Sequencing and coverage information are provided in Supplementary Table 2.

### SNV Calling and Somatic Mutation Detection

SNVs were identified using a combination of published and local pipelines (8, 12, 13) as described in Supplementary Material. Somatic point mutations were annotated using ANNOVAR (14).

### ITGH and MATH Score

Significant changes in allele frequencies were used as a surrogate for changes in clonal structure and were detected using exact binomial tests. False discovery rate (FDR) was calculated using the p.adjust R function to correct for multiple-testing (15). ITGH was measured using the mutant allele tumor heterogeneity (MATH) score (16, 17). The MATH score is a quantitative measure of ITGH based on the Mutant Allele Frequency (MAF) distribution. MATH scores were calculated as the width ratio to the center of MAFs' distribution for somatic point mutations present within individual tumors. Due to the presence of genetically distinct cellular populations, heterogeneous tumors exhibit a broader allele frequency distribution compared to homogeneous tumors and higher scores. The MATH score is the most cost-effective method to compare ITGH among different tumors and to monitor global changes in ITGH (16–21).

### Mutational Spectrum and Signatures

Mutational spectrum and signature analyses were performed, according to a previously published pipeline (22) and are detailed in Supplementary Material.

### Gene Set Enrichment Analysis

Gene set enrichment analysis (GSEA) was performed using the Molecular Signatures Database v5.0 (MSigDB) as detailed in Supplementary Material (23).

## Results

### ITGH in Rectal Tumors Is Associated With Disease Stage and Progression

**Figure 1:**
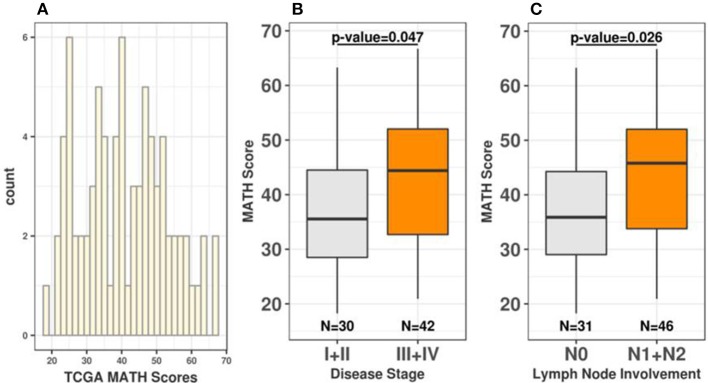
Rectal tumors exhibit continuous variability in ITGH. **(A)** Distribution of MATH scores among 79 rectal cancers from TCGA. **(B)** Distribution of MATH scores according to disease stage (Wilcoxon test *p*-value=0.047 for I+II vs. III+IV comparisons). **(C)** Distribution of MATH scores according to lymph node involvement (Wilcoxon test *p* = 0.026 for N0 vs. N1+N2).

To expand the characterization of ITGH in rectal tumors, we used WES data from 79 non-treated rectal tumors obtained from TCGA (Supplementary Figure 1). Since multi-region WES data was not available for TCGA samples, we used the MATH score to measure ITGH in these samples (16–21). Rectal tumors exhibit remarkable variability in ITGH, with MATH scores ranging from 18.2 to 66.7 (median = 40.1; mean = 41; first quartile = 31.1; third quartile = 49.8; Figure 1A). We also observed a significant positive association between MATH values, disease stage (Stage I/II median of 35.54 vs. stage III/IV median of 44.39, *p* = 0.047, Wilcoxon test, Figure 1B) and lymph node metastases (N0 median of 35.87 vs. N1+N2 median of 45.79, *p* = 0.026, Wilcoxon paired test, Figure 1C).

The MATH score is a simple and cost-effective method to measure ITGH that shows little influence of copy number variations (CNVs) and provides a first-order correction for the presence of contaminating normal tissue in tumor samples (16–21). To determine the influence of CNVs in our analysis we examined the correlation between MATH scores and total number of CNVs in all 79 individual tumors. As shown in Supplementary Figure 2, there is no significant correlation between higher MATH scores and aberrant CNV profiles in rectal tumors (cor = −0.03, *p* = 0.8, Pearson correlation). Likewise, we did not observe a significant association between the total number of CNVs, disease stage (Stage I/II median of 13.5 vs. stage III/IV median of 17.0, Wilcoxon test, *p* = 0.53, Supplementary Figure 2) or lymph node metastases (N0 median of 13 vs. N1+N2 median of 17, Wilcoxon paired test *p* = 0.42, Supplementary Figure 2). We also evaluated the impact of tumor sample purity in our results by analyzing the correlation between MATH scores and sample purity information provided for all 79 TGCA samples. As shown in Supplementary Figure 3, there is no significant correlation between MATH scores and tumor sample purity (cor = −0.037, *p* = 0.75, Pearson correlation). Therefore, non-treated rectal tumors exhibit a remarkable variability in ITGH, which is not significantly influenced by underlying somatic CNVs and tumor sample purity and is significantly associated with disease stage and lymph node metastases.

### Effects of nCRT in Normal Adjacent Mucosa

To evaluate the iatrogenic effect of nCRT, we compared the mutational landscape of a matched set of peripheral blood cells collected before nCRT (BC), tumor adjacent colonic mucosa exposed to nCRT (Nrx) and pre (PRE-T) and post-treatment (POST-T) tumor samples derived from a single rectal cancer patient.

**Figure 2:**
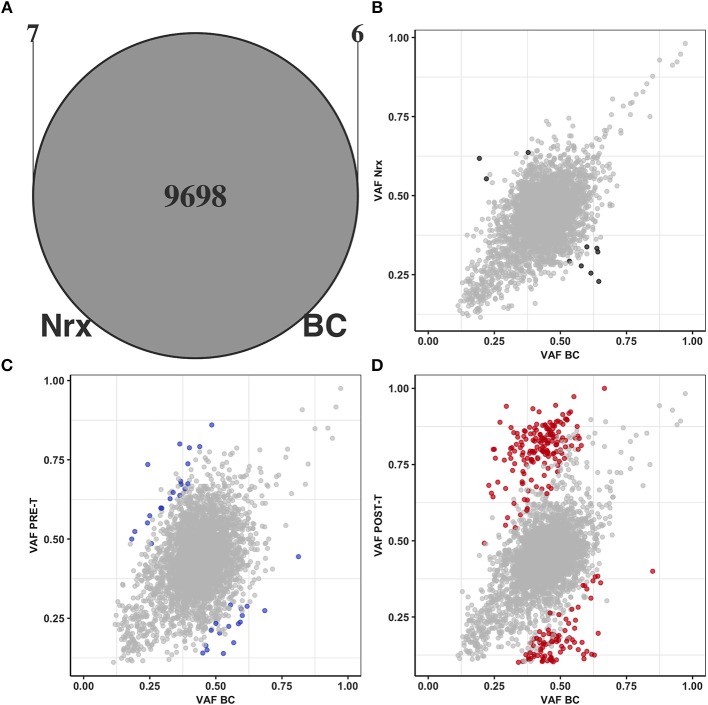
nCRT does not introduce novel somatic mutations nor affects normal tissue genetic heterogeneity. **(A)** Overlap between SNVs present in BC (*n* = 9,704) and in Nrx (*n* = 9,705). Comparisons of allele frequencies from SNVs shared by BC, Nrx, PRE-T, and POST-T samples: **(B)** BC vs. Nrx, **(C)** BC vs. PRE-T, and **(D)** BC vs. POST-T. Significant variations in allele frequencies are highlighted in black, blue, and red, respectively (*p* < 0.05; Binomial test, Bonferroni adjusted).

We first compared the total set of single nucleotide variants (SNVs) detected in BC and Nrx to determine if nCRT could introduce novel somatic mutations in the irradiated rectal colonic mucosa. As expected, since both samples are derived from the same patient, the majority of the SNVs (99.87%, 9,698/9,711) was shared between both samples and only a very small fraction of SNVs is exclusively detected in BC (0.06%, 6/9,711) or in Nrx sample (0.07%, 7/9,705) (Figure 2A). We next searched for significant variations in allele frequencies of SNVs shared between BC and NrX samples. PRE-T and POST-T samples were used as positive controls for allele frequency variations. Significant variations in allele frequencies were observed in the comparison between BC and POST-T (Figure 2D) and, to a lesser extent, in the comparison between BC and PRE-T (Figure 2C). In contrast, only 10 variants presented significant variations in allele frequencies between BC and Nrx (Figure 2B). None of these variants occurred in regions involved in V(D)J recombination but they are dispersed throughout the genome. Also, we observed a significant direct correlation of allele frequencies for SNVs present in both BC and NRX samples (*R*^2^ = 0.91; *p* < 2 × 10 – 16, Pearson Correlation test, Figure 2B). Although these results need to be validated using a larger number of matched samples, they indicate that nCRT *per se* does not seem to introduce detectable novel somatic mutations or copy number variations in the irradiated mucosa.

### ITGH Increases After nCRT

To address the effect of nCRT on the clonal structure of rectal tumors, we generated WES data from 7 matched PRE-T and POST-T samples (Supplementary Table 2). PRE-T and POST-T samples presented a median of 133 (min. 42, max. 341) and 83 (min. 50, max. 676) somatic point mutations, respectively (Supplementary Table 3). No significant difference in the number of mutations between PRE-T and POST-T tumors was observed (Wilcoxon test, *p* = 0.9). We also did not observe significant alterations in the spectrum of DNA base changes between PRE-T and POST-T samples (Supplementary Figure 4) and we were unable to detect a DNA damage mutational signature in POST-T samples (Supplementary Figure 5). Overall, the most frequent mutations observed in our cohort are also consistent with results reported by TCGA (Supplementary Table 4) (24). On average, only 20% (min. 10%—max. 32%) of the somatic mutations were shared between PRE-T and POST-T samples, with MAF correlations between matched samples ranging from *R*^2^ = 0.025–0.393 (Supplementary Table 3).

**Figure 3:**
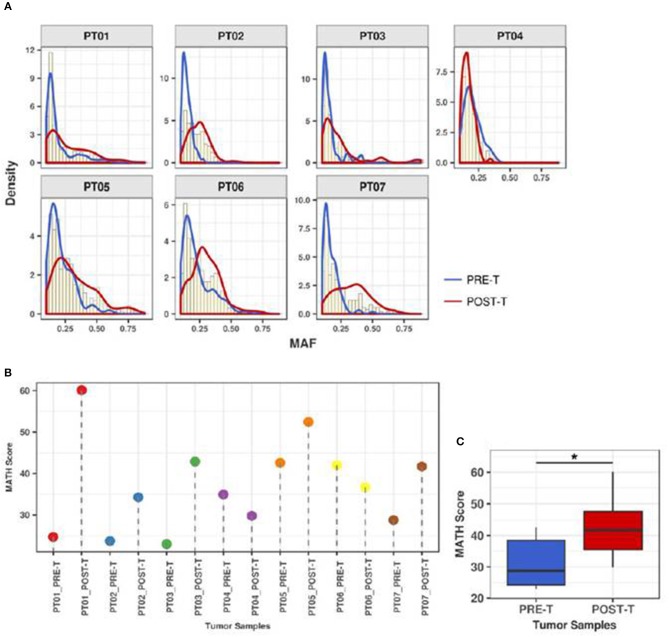
ITGH increases after nCRT. **(A)** MAF distributions for PRE-T (blue), POST-T (red), and total samples (yellow histograms) from 7 patients with rectal cancer presenting incomplete clinical response to nCRT (PT01-PT07). **(B)** MATH scores for PRE-T and POST-T samples. **(C)** Comparison between MATH scores distribution in PRE-T and POST-T samples from 7 patients with rectal cancer (*p = 0.04; paired Wilcoxon Signed-Rank Test).

To quantify the effect of nCRT on the clonal structure of rectal tumors, we next determined MAF distributions and calculated MATH scores for the 7 matched PRE-T and POST-T samples (Figures 3A,B). Median MAF and MATH scores varied from 0.13 to 0.33 and from 23 to 60.1 among all 14 samples, respectively (Supplementary Table 3). Overall MATH scores were significantly higher in POST-T samples compared to PRE-T samples (median 41.7 vs. 28.8, *p* = 0.04, Wilcoxon paired test, Figure 3C). Noteworthy, five out of the seven tumors with incomplete response to nCRT presented an increase in MATH values in POST-T sample (Figure 3B and Supplementary Table 3). This suggests that nCRT can significantly alter clonal structure in residual tumors, increasing ITGH.

### nCRT Acts as a Strong Selective Pressure

To examine if nCRT can select pre-existing tumor cell subpopulations more prone to resist to nCRT, we monitored tumor cell subpopulation dynamics before and after nCRT. Since MATH score does not allow direct enumeration of distinct tumor cell subpopulations, we monitored their dynamics by applying binomial tests to identify somatic mutations with significant changes in MAFs between PRE-T and POST-T samples (named as enriched mutations). For this analysis, we focused on 401 coding and splice site somatic mutations shared between PRE-T and POST-T samples, since they are more likely to have a deleterious impact on protein function.

**Figure 4:**
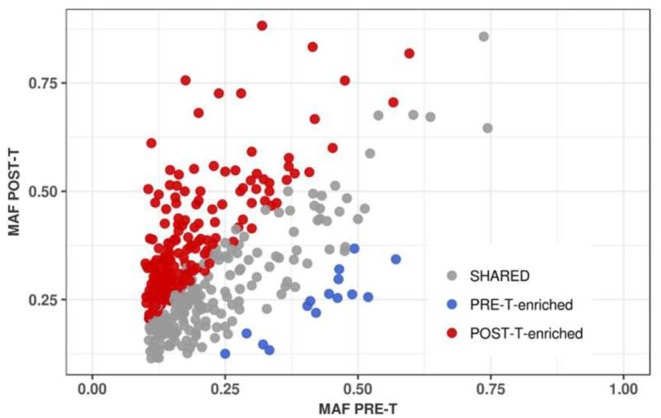
Positive selection of tumor cell subpopulations after nCRT. MAF correlation for 401 somatic point mutations shared between PRE-T and POST-T samples (R^2^ = 0.3). Enriched mutations in PRE-T (*n* = 15) and POST-T (*N* = 195) samples are highlighted in blue and red, respectively.

We identified a total of 210 somatic mutations (52.4%) enriched in PRE-T or POST-T samples (Figure 4). Mutation enrichment was validated using Sanger Sequencing (Supplementary Figure 6). Enriched mutations were significantly more frequent in POST-T compared to PRE-T samples [195/210 (92.9%) vs. 15/210 (7.1%), *p* < 0.00001^7^, Fisher's exact test, Supplementary Table 5]. Noteworthy, we observed an excess of deleterious non-synonymous mutations over neutral synonymous mutations in POST-T [136 non-synonymous (*N*) and 46 synonymous (*S*), N/S = 2.96] compared to PRE-T-enriched mutations [8 non-synonymous (N) and 5 synonymous (S), N/S = 1.6]. The observed difference, however, was not statistically significant, probably due to the small number of enriched mutations in the PRE-T samples (*p* = 0.23, Fisher's exact test).

Finally, we used GSEA to verify if nCRT could select tumor cell subpopulations more prone to resist to nCRT. In addition to the 210 enriched mutations, we found a total of 634 somatic mutations that were exclusively detected in POST-T samples. More than 59% of these POST-T specific mutations were observed in a single patient (PT02 377/634) with high tumor mutational burden. Although the presence of some of these POST-T specific mutations could be attributed to tumor topographic heterogeneity, not contemplated in the samples used for sequencing, some of these mutations could indeed result from tumor genetic instability and clonal selection during neoadjuvant therapy and were, therefore, used for GSEA. We observed that POST-T specific and POST-T enriched mutations frequently occurred in genes associated with cell cycle regulation and proliferation (mitotic spindle assembly and mitotic checkpoint gene sets) as well as with cell survival and differentiation (*K-Ras, TNF-alpha*, and Hedgehog signaling gene sets) (Supplementary Table 6). Among POST-T enriched mutations, we found non-synonymous mutations in genes associated with genetic instability and drug resistance in colorectal cancer, including mutations in the *ATM* (25, 26), *DIDO1* (27, 28), and *AKAP9* (29) (Supplementary Figure 7). Among POST-T specific mutations, we also found non-synonymous mutations in genes involved in DNA repair and apoptosis, including *ERCC6* previously associated with resistance to 5-Fluorouracil and poor prognosis (30). This suggests that nCRT may act as a strong selective pressure resulting in the selection of tumor cell subpopulations in the residual tumor that are more prone to resist to nCRT.

## Discussion

nCRT may result in significant primary tumor regression. Ultimately, tumors that achieve complete or even near-complete response may allow for organ-preserving strategies (31, 32). In this setting, even patients with early rectal cancer (cT2N0), otherwise considered for abdominal perineal resections or ultra-low anterior resections, have been considered for nCRT in order to avoid a definitive colostomy or poor anorectal function (11). However, even though patients with early stage disease are more likely to achieve a cCR, many patients will still harbor residual disease requiring surgical resection (33). In these patients, nCRT may contribute to significant increases in postoperative complications and worsening of functional anorectal outcomes (34, 35). Here, we demonstrated that nCRT may also have significant biological consequences to the residual cancer in the setting of incomplete tumor response.

In the present study, we showed that non-treated rectal tumors exhibited a remarkable variability in ITGH that is directly associated with disease stage and lymph node metastases. Studies using large tumor collections have shown that ITGH impacts clinical outcome (36, 37) and may contribute to drug resistance in different tumors (38). ITGH has been previously reported in colon (39–43) and rectal cancers (7, 8), however, for most of these studies, ITGH was determined only for a small number of tumors and the prognostic and predictive significance of ITGH for these cancers remains to be determined. Recently, the MATH score was used in two independent studies to quantify ITGH in colorectal tumors. Zhang et al. analyzed WES data from 284 colorectal tumors obtained from TCGA and 187 colorectal tumors obtained from the International Cancer Genome Consortium (ICGC) (43). The mean MATH value was 41.58 and 46.1 for the TCGA and ICGC cohorts, respectively. Similarly to our results, higher MATH scores were associated with disease stage and lymph node metastasis. Although the authors observed a significant difference in MATH scores between rectal and colon tumors (MATH = 45.9 vs. 39.96 *p* = 0.004), a separate analysis for rectal cancer was not performed and samples previously treated with nCRT were not excluded. Hardiman et al. have used the MATH score to analyze ITGH in 7 stage II/III rectal tumors, MATH scores in these tumors varied from 9.08 to 25.24 and were significantly higher in stage III tumors (7). Although further studies will be necessary to determine the prognostic significance of ITGH in rectal cancers, the strong association between ITGH and disease stage and lymph node involvement—both known predictors of survival after surgical treatment among these patients—supports a possible role for ITGH as a prognostic biomarker in rectal cancer.

Another relevant finding was that nCRT, *per se*, does not introduce detectable somatic mutations in the irradiated colonic mucosa. To the best of our knowledge, our study was the first to directly address the potentially iatrogenic effect of nCRT. The use of treatment-exposed normal colonic mucosa was critical to distinguish treatment-induced mutations from those arising from tumor genetic instability and positive clonal selection after treatment exposure. Until present, few studies have indirectly addressed the impact of radiation and chemotherapy by comparing mutational landscapes of matched PRE-T and POST-T samples (44–47). Although post-treatment mutation spectrum shifts have been reported for esophageal adenocarcinoma following platinum-based neoadjuvant chemotherapy (45), WES of matched anal squamous cell carcinomas before and after chemoradiation revealed a similar number of somatic mutations and a similar pattern of DNA substitutions in pre and post-treatment tumors (47).

The most relevant finding of the present study is that MATH scores are significantly higher in POST-T compared to PRE-T samples. This suggests that nCRT can significantly affect ITGH in residual tumors. Significant alterations in ITGH have been reported for esophageal adenocarcinoma after exposure to neoadjuvant chemotherapy. Murugaesu et al. found that mutations in post-chemotherapy samples were rarely clonal (3%), while 50% of the somatic mutations identified prior to chemotherapy were clonal (45). Findlay et al. observed a variety of clonal behaviors in esophageal tumors after chemotherapy, including samples showing little changes in clonal composition and samples with marked differences in the clonal architecture after therapy (44). Most importantly, in both studies, there was a significant association between ITGH and response to neoadjuvant chemotherapy. More recently, marked clonal landscape remodeling has also been described for hormone-positive breast cancer exposed to neoadjuvant aromatase inhibitor treatment, but no associations between ITGH and treatment response were established (48). By comparing PRE-T and POST-T samples, we observed a significant overall increase in ITGH. Five out 7 patients presented a significant increase in ITGH. Interestingly, tumor regression in these 5 samples was minimal (20–30% tumor regression). In contrast one of the 2 patients showing minimal changes in ITGH presented the most significant tumor response (70% tumor regression). Even though these results could indicate a possible association between ITGH and tumor response to nCRT, the limited size of our cohort, and the fact that we have only analyzed tumors in the setting of incomplete response to nCRT, did not allow us to fully explore this association in the present work.

Finally, we monitored tumor cell subpopulation dynamics during nCRT by identifying enriched somatic mutations with significant changes in allele frequencies between PRE-T and POST-T samples. Enriched mutations were more frequently found in POST-T samples. We also observed higher proportion of potentially deleterious mutations in these samples. Enriched mutations in POST-T samples were frequently present among genes involved in DNA damage repair, genetic instability, cell cycle regulation, proliferation, survival, and differentiation (25–29). All these molecular pathways have been shown to contribute to chemoradiotherapy resistance to colorectal tumor cells, suggesting that nCRT may result in tumors more aggressive than their baseline counterparts in the setting of incomplete response. Clonal evolution in response to neoadjuvant therapy has been previously studied in breast, esophageal, and anal squamous cell carcinomas (44, 45, 47, 48). Together with our study, these studies indicate that neoadjuvant therapy can profoundly affect tumor clonal architecture by promoting significant changes in the frequency of somatic mutations owing to the outgrowth of subclones with selective growth advantages in the residual tumor.

There are some limitations to this study that should be considered for the interpretation of our results and prevent the immediate application of our findings in clinical practice. First, we used a single set of matched samples in the analysis of the iatrogenic effect of nCRT in the normal colonic mucosa. Confirmation of our findings in larger cohorts is definitively necessary. Also, we cannot exclude the possibility that our sequencing strategy was not sensitive enough to detect novel somatic alterations, present in individual cells in the normal adjacent irradiated mucosa, which have not expanded significantly in the sampled population. Second, although we observed a significant variation in ITGH between PRE-T and POS-T samples, these observations were also based on a limited number of matched samples and in a single tumor region. We and others have described significant topographical intratumor heterogeneity in rectal cancer, and therefore, the impact of nCRT in ITGH and clonal selection in rectal tumors needs further evaluation. Most importantly, in the present work, we monitored tumor cell population dynamics by identifying “enriched mutations” with significant changes in MAFs between PRE-T and POST-T samples. Apart from a subclonal distribution and the presence of local somatic CNVs, the observed MAF at a specific locus is also directly influenced by tumor sample purity. Therefore, variations in sample purity, rather than in subclonal composition, could result in significant MAF differences between PRE-T and POST-T samples and consequently influence our analysis of tumor cell subpopulation dynamics before and after nCRT. In the present work, all tumor samples were microdissected by an experienced pathologist to enrich for tumor purity and minimize this possibility. Tumor sections were required to contain at least 80% tumor cell nuclei with <20% necrosis for inclusion in the study.

In conclusion, nCRT *per se* does not seem to introduce novel somatic mutations in the irradiated normal rectal mucosa. Instead, nCRT may drive a marked clonal selection in residual rectal tumors. This results in frequent increases in ITGH in residual cancers when compared to their baseline counterparts, which are driven by significant alterations in the frequency of biologically relevant mutations in genes associated with response to nCRT. The risk of more heterogeneous residual tumors leading to more biologically aggressive cancers may constitute a potential disadvantage of nCRT among incomplete responders. This may be particularly relevant among patients with early stage disease considering nCRT solely for the purpose of achieving cCR and organ-preservation. Future studies should address the oncological impact of significant ITGH increase after nCRT and incomplete response in rectal cancer.

## Data Availability Statement

Sequence data has been deposited at the European Genome-phenome Archive (EGA, http://www.ebi.ac.uk/ega/), which is hosted by the EBI, under accession number EGAS00001003250.

## Ethics Statement

This study was approved by the Ethics Committee of Hospital Alemão Oswaldo Cruz, São Paulo, Brazil (reference number 19/08) and was conducted in accordance with the Declaration of Helsinki. Patients provided written informed consent for tumor sample collection and study participation.

## Author Contributions

AC and RP designed the study. FB performed experiments and acquired data. AC, BC, CM, ED, FS, FB, PG, and RP analyzed and interpreted data. RP, AH-G, GS, and BV recruited patients and acquired samples for analysis. AC, RP, and PG supervised the study. AC, AH-G, PG, and RP acquired funding for the study. AC, CM, FB, and RP prepared the manuscript. AC, AH-G, BC, CM, ED, FB, PG, and RP performed critical revision of the manuscript. All authors read and approved the final manuscript.

### Conflict of Interest

The authors declare that the research was conducted in the absence of any commercial or financial relationships that could be construed as a potential conflict of interest.

## References

[1] Sauer R Becker H Hohenberger W Rödel C Wittekind C Fietkau R. Preoperative versus postoperative chemoradiotherapy for rectal cancer. N Engl J Med. (2004) 351:1731–40. 10.1056/NEJMoa040694 15496622

[2] Kosinski L. Shifting concepts in rectal cancer management. CA Cancer J Clin. (2012) 62:173–202. 10.3322/caac.21138 22488575

[3] McGranahan N Swanton C. Clonal heterogeneity and tumor evolution: past, present, and the future. Cell. (2017) 168:613–28. 10.1016/j.cell.2017.01.018 28187284

[4] Ding L Raphael BJ Chen F Wendl MC. Advances for studying clonal evolution in cancer. Cancer Lett. (2013) 340:212–9. 10.1016/j.canlet.2012.12.028 23353056 PMC3783624

[5] Gerlinger M Swanton C. How Darwinian models inform therapeutic failure initiated by clonal heterogeneity in cancer medicine. Br J Cancer. (2010) 103:1139–43. 10.1038/sj.bjc.6605912 20877357 PMC2967073

[6] Swanton C. Intratumor heterogeneity: evolution through space and time. Cancer Res. (2012) 72:4875–82. 10.1158/0008-5472.CAN-12-2217 23002210 PMC3712191

[7] Hardiman KM Ulintz PJ Kuick RD Hovelson DH Gates CM Bhasi A. Intra-tumor genetic heterogeneity in rectal cancer. Lab Investig. (2016) 96:4–15. 10.1038/labinvest.2015.131 26568296 PMC4695247

[8] Bettoni F Masotti C Habr-Gama A Correa BR Gama-Rodrigues J Vianna MR. Intratumoral genetic heterogeneity in rectal cancer: are single biopsies representative of the entirety of the tumor? Ann Surg. (2017) 265:e4–6. 10.1097/SLA.0000000000001937 27479130

[9] Cerami E Gao J Dogrusoz U Gross BE Sumer SO Aksoy BA. The cBio cancer genomics portal: an open platform for exploring multidimensional cancer genomics data. Cancer Discov. (2012) 2:401–4. 10.1158/2159-8290.CD-12-0095 22588877 PMC3956037

[10] Gao J Aksoy BA Dogrusoz U Dresdner G Gross B Sumer SO. Integrative analysis of complex cancer genomics and clinical profiles using the cBioPortal. Sci Signal. (2013) 6:pl1. 10.1126/scisignal.2004088 23550210 PMC4160307

[11] Habr-Gama A São Julião GP Vailati BB Sabbaga J Aguilar PB Fernandez LM. Organ preservation in cT2N0 rectal cancer after neoadjuvant chemoradiation therapy: the impact of radiation therapy dose-escalation and consolidation chemotherapy. Ann Surg. (2017) 269:102–7. 10.1097/SLA.0000000000002447 28742703

[12] Donnard E Asprino PF Correa BR Bettoni F Koyama FC Navarro FCP. Mutational analysis of genes coding for cell surface proteins in colorectal cancer cell lines reveal novel altered pathways, druggable mutations and mutated epitopes for targeted therapy. Oncotarget. (2014) 5:9199–213. 10.18632/oncotarget.2374 25193853 PMC4253428

[13] Torrezan GT Ferreira EN Nakahata AM Barros BDF Castro MTM Correa BR. Recurrent somatic mutation in DROSHA induces microRNA profile changes in Wilms tumour. Nat Commun. (2014) 5:4039. 10.1038/ncomms5039 24909261 PMC4062040

[14] Wang K Li M Hakonarson H. ANNOVAR: functional annotation of genetic variants from high-throughput sequencing data. Nucleic Acids Res. (2010) 38:e164. 10.1093/nar/gkq603 20601685 PMC2938201

[15] Benjamini Y Yekutieli D The control of the false discovery rate in multiple testing under depencency. Ann Stat. (2001) 29:1165–88. 10.1214/aos/1013699998

[16] Mroz EA Tward ADM Hammon RJ Ren Y Tward ADM Hammon RJ. Intra-tumor genetic heterogeneity and mortality in head and neck cancer: analysis of data from the cancer genome atlas. PLoS Med. (2015) 12:e1001786. 10.1371/journal.pmed.1001786 25668320 PMC4323109

[17] Mroz EA Tward AD Pickering CR Myers JN Ferris RL Rocco JW. High intratumor genetic heterogeneity is related to worse outcome in patients with head and neck squamous cell carcinoma. Cancer. (2013) 119:3034–42. 10.1002/cncr.28150 23696076 PMC3735618

[18] Rocco JW. Mutant Allele Tumor Heterogeneity (MATH) and head and neck squamous cell carcinoma. Head Neck Pathol. (2015) 9:1–5. 10.1007/s12105-015-0617-1 25804377 PMC4382477

[19] Ma D Jiang YZ Liu XY Liu YR Shao ZM. Clinical and molecular relevance of mutant-allele tumor heterogeneity in breast cancer. Breast Cancer Res Treat. (2017) 162:39–48. 10.1007/s10549-017-4113-z 28093659

[20] Rajput A Bocklage T Greenbaum A Lee JH Ness SA. Mutant-allele tumor heterogeneity scores correlate with risk of metastases in colon cancer. Clin Colorectal Cancer. (2016) 16:e165–70. 10.1016/j.clcc.2016.11.004 28073683 PMC5441963

[21] Mroz EA Rocco JW. MATH, a novel measure of intratumor genetic heterogeneity, is high in poor-outcome classes of head and neck squamous cell carcinoma. Oral Oncol. (2013) 49:211–5. 10.1016/j.oraloncology.2012.09.007 23079694 PMC3570658

[22] Gehring JS Fischer B Lawrence M Huber W. SomaticSignatures: inferring mutational signatures from single-nucleotide variants. Bioinformatics. (2015) 31:3673–5. 10.1101/010686 26163694 PMC4817139

[23] Subramanian A Tamayo P Mootha VK Mukherjee S Ebert BL Gillette MA. Gene set enrichment analysis: a knowledge-based approach for interpreting genome-wide expression profiles. Proc Natl Acad Sci USA. (2005) 102:15545–50. 10.1073/pnas.0506580102 16199517 PMC1239896

[24] The Cancer Genome Atlas Network Comprehensive molecular characterization of human colon and rectal cancer. Nature. (2012) 487:330–7. 10.1038/nature11252 22810696 PMC3401966

[25] Zhou Y Wan G Spizzo R Ivan C Mathur R Hu X. miR-203 induces oxaliplatin resistance in colorectal cancer cells by negatively regulating ATM kinase. Mol Oncol. (2014) 8:83–92. 10.1016/j.molonc.2013.09.004 24145123 PMC4124530

[26] Yao J Huang A Zheng X Liu T Lin Z Zhang S. 53BP1 loss induces chemoresistance of colorectal cancer cells to 5-fluorouracil by inhibiting the ATM–CHK2–P53 pathway. J Cancer Res Clin Oncol. (2017) 143:419–31. 10.1007/s00432-016-2302-5 27838786 PMC11819077

[27] Rojas AM Sanchez-Pulido L Fütterer A Van Wely KHM Martinez-A C Valencia A. Death inducer obliterator protein 1 in the context of DNA regulation: Sequence analyses of distant homologues point to a novel functional role. FEBS J. (2005) 272:3505–11. 10.1111/j.1742-4658.2005.04759.x 16008551

[28] Trachana V van Wely KHM Guerrero AA Fütterer A Martínez-A C. Dido disruption leads to centrosome amplification and mitotic checkpoint defects compromising chromosome stability. Proc Natl Acad Sci USA. (2007) 104:2691–6. 10.1073/pnas.0611132104 17299043 PMC1815243

[29] Jo YS Kim MS Yoo NJ Lee SH. Frameshift mutations of AKAP9 gene in gastric and colorectal cancers with high microsatellite instability. Pathol Oncol Res. (2016) 22:587–92. 10.1007/s12253-016-0042-0 26786868

[30] Zhao Z Zhang G Li W. Elevated expression of ERCC6 confers resistance to 5-fluorouracil and is associated with poor patient survival in colorectal cancer. DNA Cell Biol. (2017) 36:781–6. 10.1089/dna.2017.3768 28665687

[31] van der Valk MJM Hilling DE Bastiaannet E Meershoek-Klein KE Beets GL Figueiredo NL. Long-term outcomes of clinical complete responders after neoadjuvant treatment for rectal cancer in the International Watch & Wait Database (IWWD): an international multicentre registry study. Lancet. (2018) 391:2537–45. 10.1016/S0140-6736(18)31078-X 29976470

[32] Rullier E Rouanet P Tuech JJ Valverde A Lelong B Rivoire M. Organ preservation for rectal cancer (GRECCAR 2): a prospective, randomised, open-label, multicentre, phase 3 trial. Lancet. (2017) 390:469–79. 10.1016/S0140-6736(17)31056-5 28601342

[33] Chadi SA Malcomson L Ensor J Riley RD Vaccaro CA Rossi GL. Factors affecting local regrowth after watch and wait for patients with a clinical complete response following chemoradiotherapy in rectal cancer (InterCoRe consortium): an individual participant data meta-analysis. Lancet Gastroenterol Hepatol. (2018) 3:825–36. 10.1016/S2468-1253(18)30301-7 30318451

[34] Loos M Quentmeier P Schuster T Nitsche U Gertler R Keerl A. Effect of preoperative radio(chemo)therapy on long-term functional outcome in rectal cancer patients: a systematic review and meta-analysis. Ann Surg Oncol. (2013) 20:1816–28. 10.1245/s10434-012-2827-z 23269466

[35] Battersby NJ Juul T Christensen P Janjua AZ Branagan G Emmertsen KJ. Predicting the risk of bowel-related quality-of-life impairment after restorative resection for rectal cancer: a multicenter cross-sectional study. Dis Colon Rectum. (2016) 59:270–80. 10.1097/DCR.0000000000000552 26953985

[36] Morris LGT Riaz N Desrichard A Senbabaoglu Y Hakimi AA Makarov V. Pan-cancer analysis of intratumor heterogeneity as a prognostic determinant of survival. Oncotarget. (2016) 7:10051–63. 10.18632/oncotarget.7067 26840267 PMC4891103

[37] Andor N Graham TA Jansen M Xia LC Aktipis CA Petritsch C. Pan-cancer analysis of the extent and consequences of intratumor heterogeneity. Nat Med. (2015) 22:105–13. 10.1038/nm.3984 26618723 PMC4830693

[38] McGranahan N Favero F de Bruin EC Birkbak NJ Szallasi Z Swanton C. Clonal status of actionable driver events and the timing of mutational processes in cancer evolution. Sci Transl Med. (2015) 7:283ra54. 10.1126/scitranslmed.aaa1408 25877892 PMC4636056

[39] Sveen A Løes IM Alagaratnam S Nilsen G Høland M Lingjærde OC. Intra-patient inter-metastatic genetic heterogeneity in colorectal cancer as a key determinant of survival after curative liver resection. PLOS Genet. (2016) 12:e1006225. 10.1371/journal.pgen.1006225 27472274 PMC4966938

[40] Kim TM Jung SH An CH Lee SH Baek IP Kim MS. Subclonal genomic architectures of primary and metastatic colorectal cancer based on intratumoral genetic heterogeneity. Clin Cancer Res. (2015) 21:4461–72. 10.1158/1078-0432.CCR-14-2413 25979483

[41] Braxton DR Zhang R Morrissette JD Loaiza-Bonilla A Furth EE. Clinicopathogenomic analysis of mismatch repair proficient colorectal adenocarcinoma uncovers novel prognostic subgroups with differing patterns of genetic evolution. Int J Cancer. (2016) 139:1546–56. 10.1002/ijc.30196 27194209

[42] Jesinghaus M Pfarr N Kloor M Endris V Tavernar L Muckenhuber A. Genetic heterogeneity in synchronous colorectal cancers impacts genotyping approaches and therapeutic strategies. Genes Chromosom Cancer. (2016) 55:26877. 10.1002/gcc.22330 26650777

[43] Zhang J Yan S Liu X Gan L Wu Z Gong Y. Gender-related prognostic value and genomic pattern of intra-tumor heterogeneity in colorectal cancer. Carcinogenesis. (2017) 38:837–46. 10.1093/carcin/bgx046 28531253 PMC5862243

[44] Findlay JM Castro-Giner F Makino S Rayner E Kartsonaki C Cross W. Differential clonal evolution in oesophageal cancers in response to neo-adjuvant chemotherapy. Nat Commun. (2016) 7:11111. 10.1038/ncomms11111 27045317 PMC4822033

[45] Murugaesu N Wilson GA Birkbak NJ Watkins TBK McGranahan N Kumar S. Tracking the genomic evolution of esophageal adenocarcinoma through neoadjuvant chemotherapy. Cancer Discov. (2015) 5:821–32. 10.1158/2159-8290.CD-15-0412 26003801 PMC4529488

[46] Faltas BM Prandi D Tagawa ST Molina AM Nanus DM Sternberg C. Clonal evolution of chemotherapy-resistant urothelial carcinoma. Nat Genet. (2016) 48:1490–9. 10.1038/ng.3692 27749842 PMC5549141

[47] Mouw KW Cleary JM Reardon B Pike J Braunstein LZ Kim J. Genomic evolution after chemoradiotherapy in anal squamous cell carcinoma. Clin Cancer Res. (2017) 23:3214–22. 10.1158/1078-0432.CCR-16-2017 27852700 PMC5433927

[48] Miller CA Gindin Y Lu C Griffith OL Griffith M Shen D. Aromatase inhibition remodels the clonal architecture of estrogen-receptor-positive breast cancers. Nat Commun. (2016) 7:12498. 10.1038/ncomms12498 27502118 PMC4980485

