# Supplementary material for: The Effects of Neoadjuvant Chemoradiation in Locally Advanced Rectal Cancer—The Impact in Intratumoral Heterogeneity
Source: Front Oncol. 2019 Sep 27;9:974. doi: 10.3389/fonc.2019.00974 (PMC6776613; doi:10.3389/fonc.2019.00974)
Supplement: Supplementary file 7 [file Data_Sheet_1.PDF]

Supplementary Materials and Methods

1 TCGA Data

We selected 79 primary rectal tumors from the TCGA colorectal cancer cohort(1,2). Samples from patients who underwent neoadjuvant therapy and presented less than 20 somatic mutations after filtering steps were removed from our analysis. Tumor-specific SNVs (somatic mutations) were filtered to ensure they were reported by at least 3 tumor reads and covered by at least 30 sequencing reads. MAF files for selected TCGA samples were downloaded from Broad Institute GDACFirehose MAF Dashboard (https://confluence.broadinstitute.org/display/GDAC/MAF+Dashboard). Level 3 CNV data used in this analysis was downloaded from: http://firebrowse.org/?cohort=READ&download_dialog=true. Corresponding clinical data for selected samples were downloaded from cBioPortal for Cancer Genomics (http://www.cbioportal.org/). Clinical, pathological and mutational data for all 79 primary rectal tumors are provided in Supplementary Table 1.

2 Tumor and Blood Samples

Fresh tumor samples were collected at diagnosis by endoscopic biopsy (PRE-T samples) and during surgical removal of the residual tumors after at least 12 weeks from nCRT completion (POST-T samples). Tumor samples were immediately snap-frozen in liquid nitrogen and stored at -80°C. Prior to DNA extraction, fragments were assessed for tumor purity through macrodissection. Only samples with >80% tumor cells were selected for sequencing. Tumor-adjacent normal colonic mucosa exposed to nCRT (Nrx) was also collected from one patient (PT07) during surgical removal of the residual tumor. Peripheral blood cells (BC) were collected from all patients. Genomic DNA from tumor and normal samples was extracted using standard phenol/chlorophorm extraction. Whole-exome libraries were prepared using SureSelect Human All Exon Target Enrichment kit (Agilent Technologies, Santa Clara, CA) and 75bp single-end sequences were generated on a 5500xl SOLiD sequencing platform (Thermo-Fisher Scientific, Waltham, MA). Sequencing and coverage information is provided in Supplementary Table 2.

3 SNV Calling and Somatic Mutation Detection

SNVs were identified using a combination of published and local pipelines(3–5). Briefly, genomic sequences were aligned to the human genome reference sequence (GRCh37/hg19) using BioScope (Life Technologies). Duplicated reads were removed with SAMtools(6). SAMtools mpileup and bcftools were then used to identify SNVs(6,7). Only SNVs reported by at least 3 supporting reads with mapping quality (Q) >= 20 were selected for further analyses. To minimize false calls due to sequencing and alignment errors, we excluded SNVs (i) with more than two alternative alleles, (ii) with strand bias, (iii) within regions of low mappability scores (UCSC Mappability Tracks, Alignability 75mers scores lower than 1), and (iv) within loci under allelic mapping bias(8). Somatic point mutations were identified after removing i) germline SNVs present in the matched normal samples; ii) SNVs with locus coverage <3 reads in the matched normal sample; iii) SNVs with locus coverage <30 reads in tumor samples; iv) SNVs reported in databases of known human germline variants (NHLBI Grand Opportunity Exome Sequencing Project, 1000 Genomes Project and Exome Aggregation Consortium) with minor allele frequencies higher than 0.1%; v) SNVs reported in dbSNP (release #144) but within genes with COSMIC (version 78) mutation frequency <0.5%. Recurrent somatic

mutations (present in more than one patient) were also removed if they did not occur in recognized colorectal cancer driver genes (*APC, TP53, FBXW7, NRAS, TCF7L2, BRAF, KRAS, PIK3CA, ARID1A,* and *CASP8*)(9). Somatic point mutations were annotated using ANNOVAR Gene-based Annotation(10) and the corresponding mutant allele fractions (MAFs) were determined as the proportion of reads reporting the mutant allele over the total number of reads covering the variant locus.

4 Clonal Architecture Dynamics and ITGH Analysis

Changes in tissue clonal architecture were monitored by tracking significant variations in MAFs between two matched samples for a specific SNV or somatic mutation (eg. BC vs. Nrx samples and PRE-T vs. POST-T samples). For this analysis, we only considered SNVs or somatic mutations with at least 30X coverage. Significant changes in MAFs were detected using exact binomial tests. False discovery rate (FDR) was calculated using the *p.adjust* R function to correct for multiple-testing(11). SNVs or somatic mutations with significant changes in MAFs between two matched samples (p-value<0.5 and FDR<10%) were defined as an enriched SNV or somatic mutation. ITGH was measured using the mutant allele tumor heterogeneity (MATH) score(12,13). MATH scores were calculated as the median absolute deviation divided by the median MAF of all somatic mutations detected in the sample. As previously suggested, for MATH score calculations we used somatic mutations calls with MAF of 0.1 or greater. We compared MATH scores from PRE-T and POST-T samples using the paired Wilcoxon Signed-Rank Test.

5 Mutational Spectrum and Mutational Signature Analysis

Mutational spectrum analysis was performed using coding and splicing somatic mutations identified in our matched pre and post-treatment samples. We identified mutation context based on the human reference genome (hg19) through Bioconductor's Somatic Signatures and BSGenome packages for R software (v3.2.4), according to a previously published pipeline(14). Mutational signatures were determined using the Mutation Analysis Toolkit (http://www.mutalisk.org).

6 Sanger Sequencing

Changes in MAF for a subset of enriched mutations were independently validated by PCR amplification of the mutated region using DNA from PRE-T and POST-T samples and matched BC. PCR amplicons (primers upon request) were then sequenced using Sanger protocol on an ABI 3130xl (Thermo Fisher Scientific, Waltham, MA). Sequence chromatograms were manually inspected to detect differences in peak height corresponding to the mutant allele.

7 Gene Set Enrichment Analysis

Gene set enrichment analysis (GSEA) was performed to compute the overlap between genes with POST-T specific and POST-T enriched mutations and annotated gene sets from the Molecular Signatures Database v5.0 (MSigDB), such as Hallmarks and KEGG (Kyoto Encyclopedia of Genes and Genomes) pathways(15). Overlaps were computed using HUGO gene symbols and significance was estimated using the hypergeometric distribution for (k-1, K, N-K, n), where k is the number of genes intersecting the set from MSigDB, K is the number of genes in MSigDB set, N is the total number of known gene symbols, and n is the total number of genes in the query set.

8 References

1. Cerami E, Gao J, Dogrusoz U, Gross BE, Sumer SO, Aksoy BA, et al. The cBio Cancer Genomics Portal: An open platform for exploring multidimensional cancer genomics data. *Cancer Discov* (2012) 2:401–404. doi:10.1158/2159-8290.CD-12-0095
2. Gao J, Aksoy BA, Dogrusoz U, Dresdner G, Gross B, Sumer SO, et al. Integrative analysis of complex cancer genomics and clinical profiles using the cBioPortal. *Sci Signal* (2013) 6:pl1. doi:10.1126/scisignal.2004088
3. Bettoni F, Masotti C, Habr-Gama A, Correa BR, Gama-Rodrigues J, Vianna MR, et al. Intratumoral Genetic Heterogeneity in Rectal Cancer: Are Single Biopsies representative of the entirety of the tumor? *Ann Surg* (2017) 265:e4–e6. doi:10.1097/SLA.0000000000001937
4. Donnard E, Asprino PF, Correa BR, Bettoni F, Koyama FC, Navarro FCP, et al. Mutational analysis of genes coding for cell surface proteins in colorectal cancer cell lines reveal novel altered pathways, druggable mutations and mutated epitopes for targeted therapy. *Oncotarget* (2014) 5:9199–213. doi:10.18632/oncotarget.2374
5. Torrezan GT, Ferreira EN, Nakahata AM, Barros BDF, Castro MTM, Correa BR, et al. Recurrent somatic mutation in DROSHA induces microRNA profile changes in Wilms tumour. *Nat Commun* (2014) 5:4039. doi:10.1038/ncomms5039
6. Li H, Handsaker B, Wysoker A, Fennell T, Ruan J, Homer N, et al. The Sequence Alignment/Map format and SAMtools. *Bioinformatics* (2009) 25:2078–2079. doi:10.1093/bioinformatics/btp352
7. Li H. A statistical framework for SNP calling, mutation discovery, association mapping and population genetical parameter estimation from sequencing data. *Bioinformatics* (2011) 27:2987–2993. doi:10.1093/bioinformatics/btr509
8. Panousis NI, Gutierrez-Arcelus M, Dermitzakis ET, Lappalainen T. Allelic mapping bias in RNA-sequencing is not a major confounder in eQTL studies. *Genome Biol* (2014) 15:467. doi:10.1186/s13059-014-0467-2
9. Lawrence MS, Stojanov P, Mermel CH, Robinson JT, Garraway L a, Golub TR, et al. Discovery and saturation analysis of cancer genes across 21 tumour types. *Nature* (2014) 505:495–501. doi:10.1038/nature12912
10. Wang K, Li M, Hakonarson H. ANNOVAR: functional annotation of genetic variants from high-throughput sequencing data. *Nucleic Acids Res* (2010) 38:e164. doi:10.1093/nar/gkq603
11. Benjamini Y, Yekutieli D. The control of the false discovery rate in multiple testing under depencency. *Ann Stat* (2001) 29:1165–1188. doi:10.1214/aos/1013699998
12. Mroz EA, Tward ADM, Hammon RJ, Ren Y, Tward ADM, Hammon RJ, et al. Intra-tumor Genetic Heterogeneity and Mortality in Head and Neck Cancer : Analysis of Data from The Cancer Genome Atlas. *PLoS Med* (2015) 12:1–27. doi:10.7908/C1VH5KV4
13. Mroz EA, Tward AD, Pickering CR, Myers JN, Ferris RL, Rocco JW. High intratumor genetic heterogeneity is related to worse outcome in patients with head and neck squamous cell carcinoma. *Cancer* (2013) 119:3034–3042. doi:10.1002/cncr.28150
14. Gehring JS, Fischer B, Lawrence M, Huber W. SomaticSignatures: Inferring mutational signatures from single-nucleotide variants. *Bioinformatics* (2015) 31:3673–3675. doi:10.1093/bioinformatics/btv408
15. Subramanian A, Tamayo P, Mootha VK, Mukherjee S, Ebert BL, Gillette MA, et al. Gene set enrichment analysis: A knowledge-based approach for interpreting genome-wide expression profiles. *Proc Natl Acad Sci* (2005) 102:15545–15550. doi:10.1073/pnas.0506580102
