# Supplementary material for: The Effects of Neoadjuvant Chemoradiation in Locally Advanced Rectal Cancer—The Impact in Intratumoral Heterogeneity
Source: Front Oncol. 2019 Sep 27;9:974. doi: 10.3389/fonc.2019.00974 (PMC6776613; doi:10.3389/fonc.2019.00974)
Supplement: Supplementary file 8 [file Data_Sheet_2.PDF]

Supplementary Figures

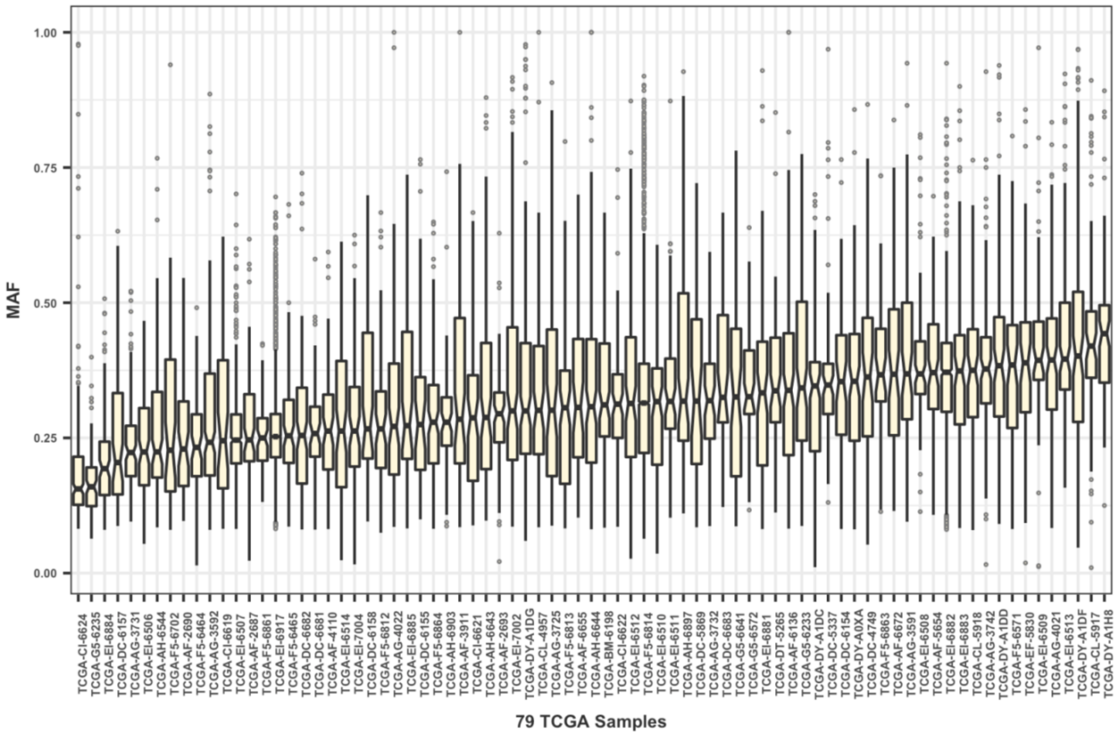

Supplementary Figure 1. Distribution of mutant allele frequencies in non-treated rectal tumors from TCGA cohort. MAF files for 79 non-treated primary rectal tumors from the TCGA colorectal cancer cohort were downloaded from Broad Institute GDAC Firehose MAF Dashboard.

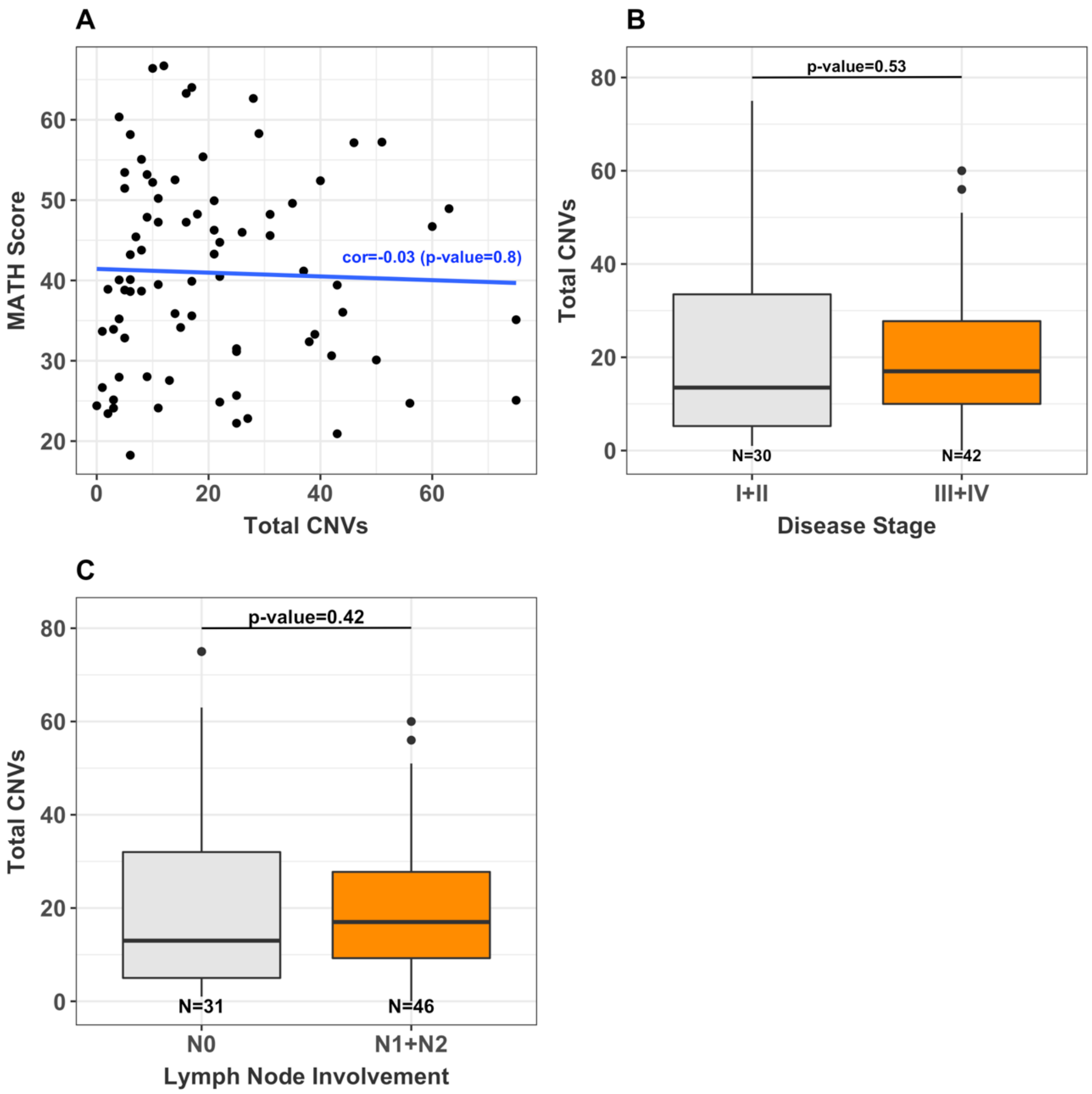

Supplementary Figure 2. Analysis of aberrant CNVs in rectal cancer. (A) Correlation analysis between MATH scores and total number of CNVs for 79 TCGA-READ tumor samples (corr=-0.03, p-value=0.8; Pearson correlation). (B) Distribution of the total number of CNVs according to disease stage (Wilcoxon test p-value=0.53 for I+II vs. III+IV comparisons). (C) Distribution of the total number of CNVs according to lymph node involvement (Wilcoxon test p-value=0.42 for N0 vs. N1+N2). Data used for this analysis is described in Supplementary Materials and Methods.

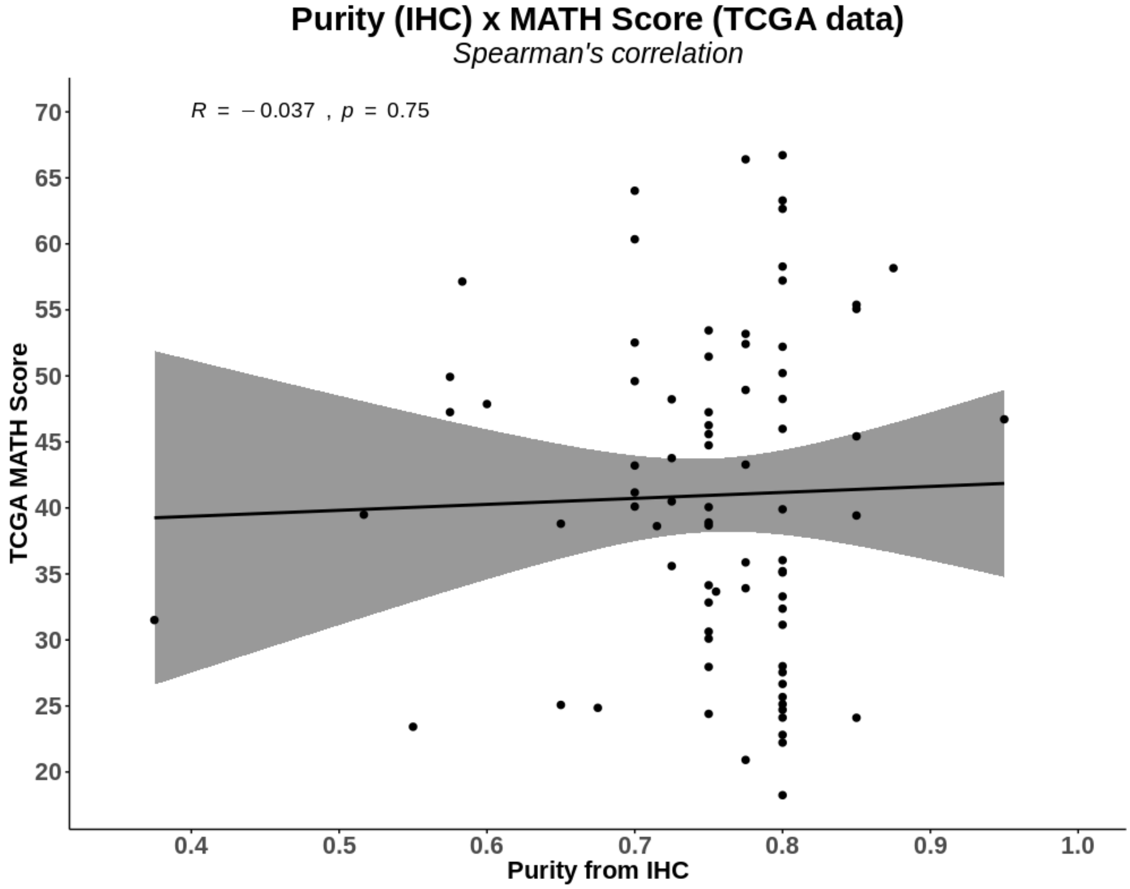

Supplementary Figure 3. MATH score and tumor sample purity. Correlation analysis between MATH scores and sample purity accessed by immunohistochemistry (IHC) for 79 TCGA-READ tumor samples (corr=-0.037, p-value=0.75; Pearson correlation).

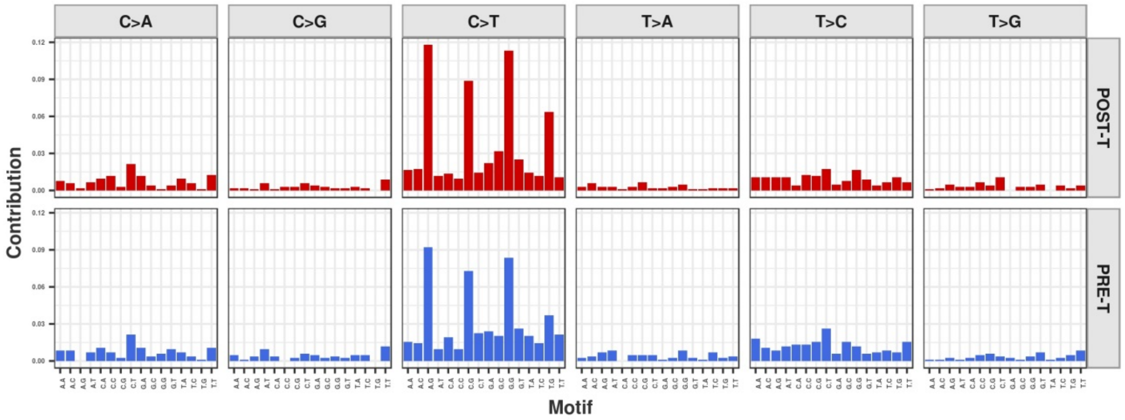

Supplementary Figure 4. Spectrum of DNA base changes in PRE-T and POST-T samples. The spectrum of somatic mutations present in PRE-T and POST-T samples is displayed using a 96-substitution classification defined by substitution type and sequence context immediately 5′ and 3′ to the mutated base. Percentages of mutations attributed to specific substitution types are represented on the *y*-axes, whereas the *x*-axes display different types of substitutions.

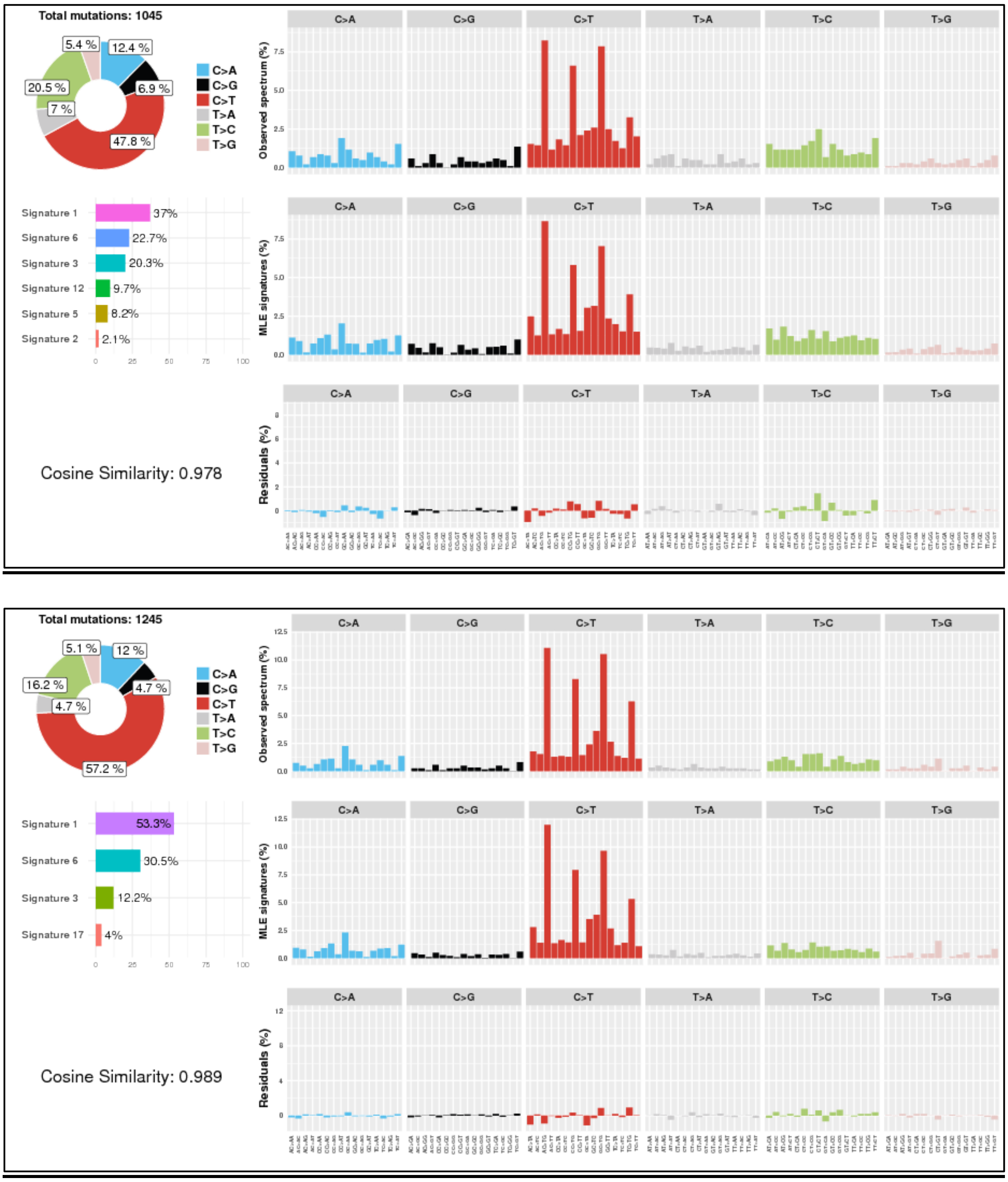

Supplementary Figure 5. Mutational spectrum and mutational signatures present in PRE-T (top) and POST-T (bottom) samples. Mutational spectrum and COSMIC mutation signatures analysis were performed with coding and splicing somatic mutations identified in our matched pre (top) and post-treatment (bottom) samples using Mutalisk.

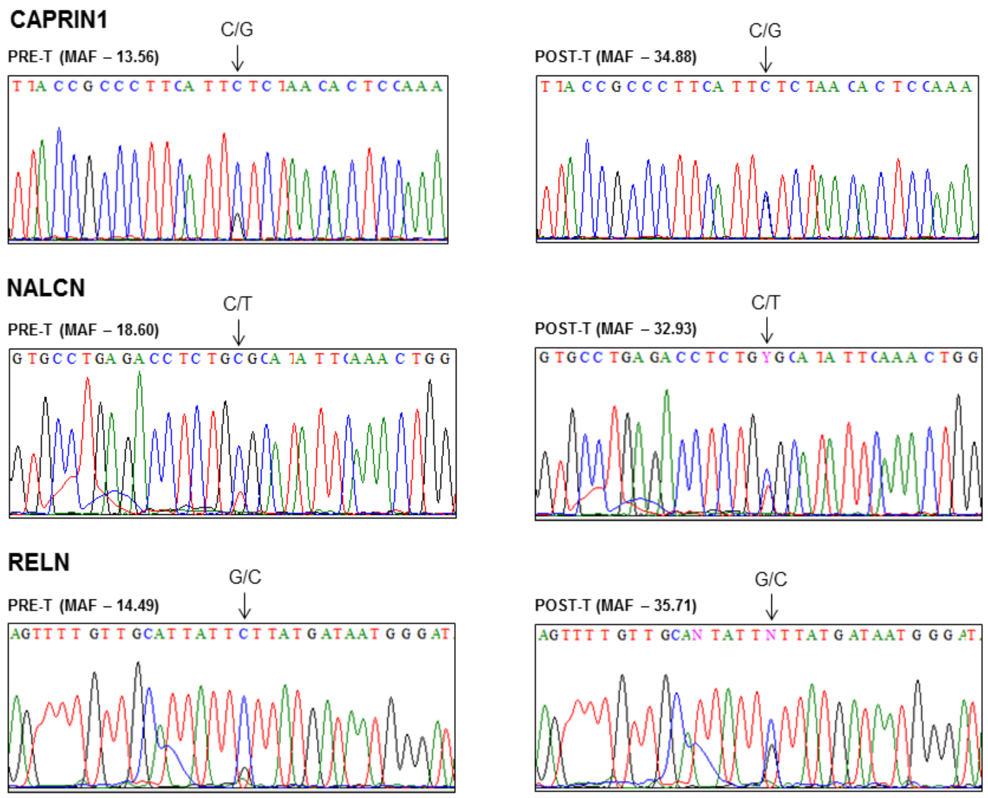

Supplementary Figure 6. Sanger sequencing validation of enriched mutations. Changes in MAFs for enriched mutations present in CAPRIN1, NALCN and RELN genes were validated by PCR amplification and Sanger sequencing of the mutated region using DNA from PRE-T and POST-T samples. Sequence chromatograms were manually inspected to detect differences in peak height corresponding to the mutant allele.

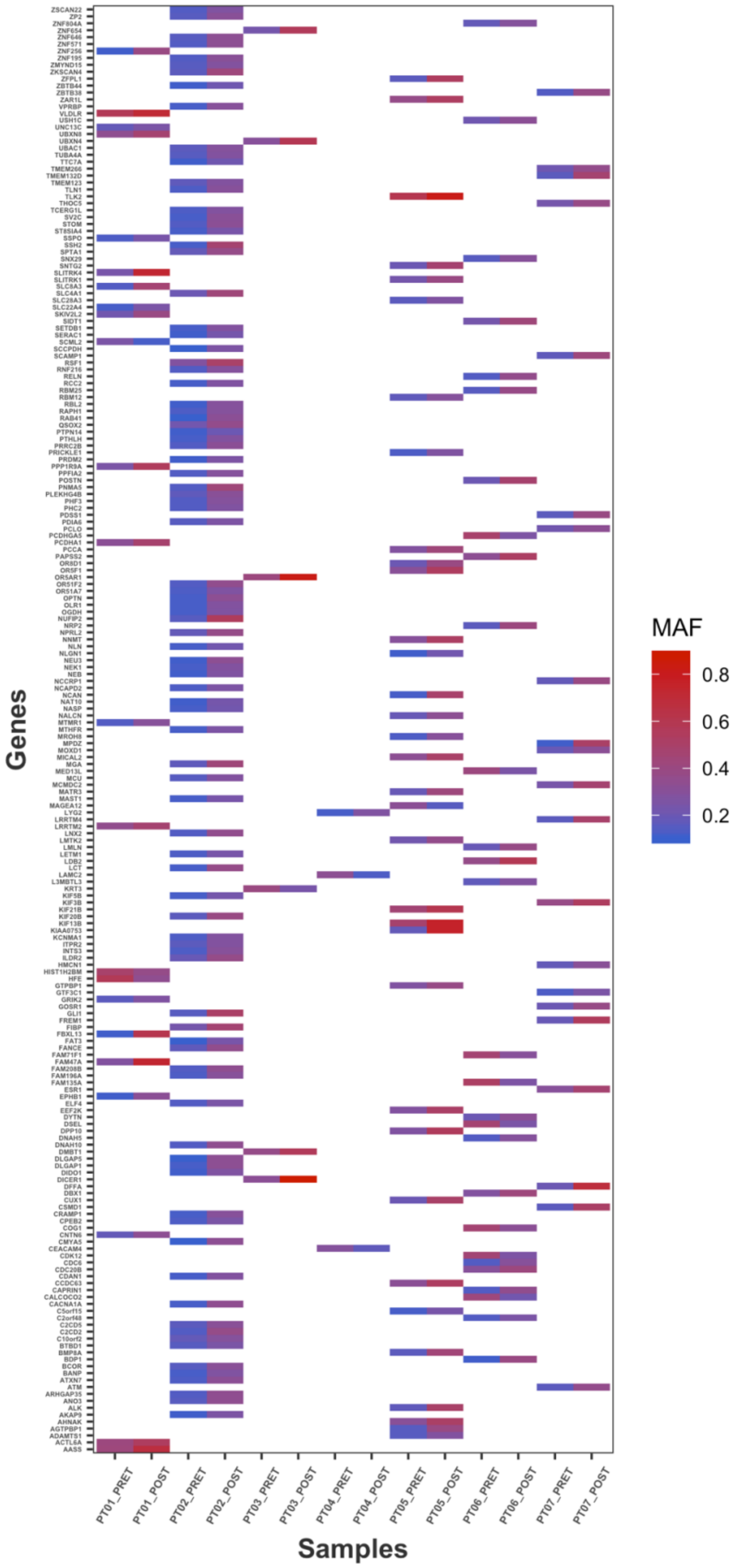

Supplementary Figure 7. Mutant allele frequencies for somatic mutations enriched in PRE-T and POST-T samples. List of somatic mutations detected in PRE-T and POST-T samples from rectal cancer patients (PT01-PT07). Mutant allele frequencies for enriched mutations in 209 mutated genes are represented using a color gradient.
